# Colistin-, cefepime-, and levofloxacin-resistant *Salmonella enterica* serovars isolated from Egyptian chicken carcasses

**DOI:** 10.1186/s12941-024-00713-3

**Published:** 2024-07-04

**Authors:** Bassant Ashraf El-Saeed, Hend Ali Elshebrawy, Amira Ibrahim Zakaria, Adel Abdelkhalek, Khalid Ibrahim Sallam

**Affiliations:** 1 Department of Food Hygiene, Safety, and Technology, Faculty of Veterinary Medicine, Mansoura University, Mansoura, 35516 Egypt; 2 Faculty of Veterinary Medicine, Badr University in Cairo (BUC), Badr City, 11829 Cairo Egypt

**Keywords:** Virulence genes, Salmonella serovars, Chicken meat, β-lactamase, Antimicrobial resistance

## Abstract

**Objectives:**

The emergence of multidrug-resistant (MDR) *Salmonella* strains, especially resistant ones toward critically important antimicrobial classes such as fluoroquinolones and third- and fourth-generation cephalosporins, is a growing public health concern. The current study, therefore, aimed to determine the prevalence, and existence of virulence genes (*invA*, *stn*, and *spvC* genes), antimicrobial resistance profiles, and the presence of β-lactamase resistance genes (*bla*_OXA_, *bla*_CTX-M1_, *bla*_SHV_, and *bla*_TEM_) in *Salmonella* strains isolated from native chicken carcasses in Egypt marketed in Mansoura, Egypt, as well as spotlight the risk of isolated MDR, colistin-, cefepime-, and levofloxacin-resistant *Salmonella* enterica serovars to public health.

**Methods:**

One hundred fifty freshly dressed native chicken carcasses were collected from different poultry shops in Mansoura City, Egypt between July 2022 and November 2022. *Salmonella* isolation was performed using standard bacteriological techniques, including pre-enrichment in buffered peptone water (BPW), selective enrichment in Rappaport Vassiliadis broth (RVS), and cultivating on the surface of xylose-lysine-desoxycholate (XLD) agar. All suspected *Salmonella* colonies were subjected to biochemical tests, serological identification using slide agglutination test, and Polymerase Chain Reaction (PCR) targeting the invasion A gene (*invA*; *Salmonella* marker gene). Afterward, all molecularly verified isolates were screened for the presence of virulence genes (*stn* and *spvC*). The antimicrobial susceptibility testing for isolated *Salmonella* strains towards the 16 antimicrobial agents tested was analyzed by Kirby–Bauer disc diffusion method, except for colistin, in which the minimum inhibition concentration (MIC) was determined by broth microdilution technique. Furthermore, 82 cefotaxime-resistant *Salmonella* isolates were tested using multiplex PCR targeting the β-lactamase resistance genes, including *bla*_OXA_, *bla*_CTX-M1_, *bla*_SHV_, and *bla*_TEM_ genes.

**Results:**

*Salmonella enterica* species were molecularly confirmed via the *invA Salmonella* marker gene in 18% (27/150) of the freshly dressed native chicken carcasses. Twelve *Salmonella* serotypes were identified among 129 confirmed *Salmonella* isolates with the most predominant serotypes were* S*. Kentucky, *S*. Enteritidis, *S*. Typhimurium, and *S*. Molade with an incidence of 19.4% (25/129), 17.1% (22/129), 17.1% (22/129), and 10.9% (14/129), respectively. All the identified *Salmonella* isolates (n = 129) were positive for both *invA* and *stn* genes, while only 31.8% (41/129) of isolates were positive for the *spvC* gene. One hundred twenty-one (93.8%) of the 129 *Salmonella*-verified isolates were resistant to at least three antibiotics. Interestingly, 3.9%, 14.7%, and 75.2% of isolates were categorized into pan-drug-resistant, extensively drug-resistant, and multidrug-resistant, respectively. The average MAR index for the 129 isolates tested was 0.505. Exactly, 82.2%, 82.2%, 63.6%, 51.9%, 50.4%, 48.8%, 11.6%, and 10.1% of isolated *Salmonella* strains were resistant to cefepime, colistin, cefotaxime, ceftazidime/clavulanic acid, levofloxacin, ciprofloxacin, azithromycin, and meropenem, respectively. Thirty-one out (37.8%) of the 82 cefotaxime-resistant *Salmonella* isolates were β-lactamase producers with the *bla*_TEM_ as the most predominant β-lactamase resistance gene, followed by *bla*_CTX-M1_ and *bla*_OXA_ genes, which were detected in 21, 16, and 14 isolates respectively).

**Conclusion:**

The high prevalence of MDR-, colistin-, cefepime-, and levofloxacin-resistant *Salmonella* serovars among *Salmonella* isolates from native chicken is alarming as these antimicrobials are critically important in treating severe salmonellosis cases and boost the urgent need for controlling antibiotic usage in veterinary and human medicine to protect public health.

**Graphical Abstract:**

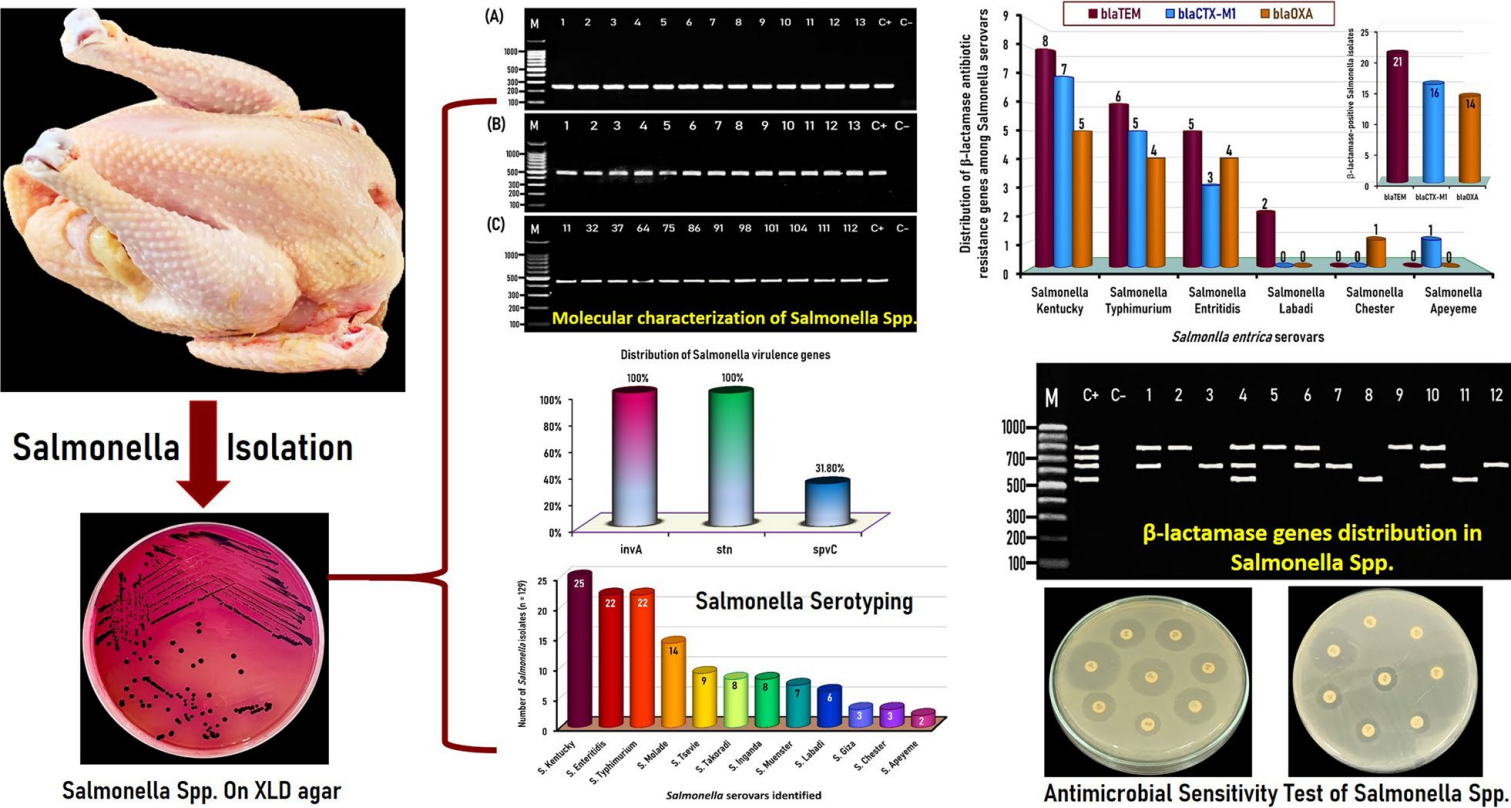

## Introduction

Chicken meat is considered one of the most consumed meats worldwide, and the average world annual production of chicken meat increased from 83.3 million metric tons in 2012 to about 103.4 million metric tons in 2023 (https://www.statista.com/statistics/237637/production-of-poultry-meat-worldwide-since-1990/). Chicken meat is widespread among Egyptian consumers across all income denominations. In Egypt, poultry meat consumption is about 1.2 billion birds per year. Furthermore, the total quantity of poultry meat consumed in Egypt increased from 1.13 million metric tons in 2016 to 1.46 million metric tons in 2020 (https://www.fao.org/faostat/en/#data/FBS).

Chicken is the leading reservoir for *Salmonella*, which is mainly present in the intestines of live birds. Chicken carcasses are contaminated mainly with *Salmonella* species owing to fecal cross-contamination, so *Salmonella* could cling to the skin of chicken carcasses and/or become stuck inside the skin feather follicles [1]. Live bird markets constitute the primary source for chicken carcass contamination by *Salmonella* species. The poor hygienic practices in live bird markets may permit the spread of foodborne pathogens to humans as live birds were housed at high intensity in cages with confined spaces until they were slaughtered and sold as freshly dressed carcasses. Chicken carcasses are contaminated mainly due to unhygienic practices during the several processing steps, such as slaughtering, scalding, plucking, evisceration, washing, and chilling. Moreover, cages, chopping boards, butchers’ hands, and knives are potential sources for contamination of chicken carcasses by *Salmonella* serotypes [2].

Nowadays, there are over 2700 *Salmonella* serotypes [3]. *Salmonella* species isolated from chicken carcasses include a wide variety of serovars, for instance, *S*. Typhimurium, *S*. Kentucky, *S*. Enteritidis, *S*. Infantis, *S*. Molade, *S*. Bargny, and *S*. Inganda [4, 5]. Although most *Salmonella* infections are mild, some can be life-threatening according to the serotype and host factors. *Salmonella* causes abundant morbidity and mortality, mostly in developing countries [6]. *S*. Typhimurium and *S*. Enteritidis caused around 75% of human salmonellosis cases, represented by fever, vomiting, diarrhea, nausea, and abdominal cramps [7].

*Salmonella* is one of the most important foodborne pathogens in humans and animals worldwide and has been widely related to foodborne outbreaks. Approximately 1.3 billion infection cases and 155,500 deaths annually worldwide are attributed to *Salmonella* [8]. In the USA, about 1.35 million infections, 26,500 hospitalizations, and 420 deaths yearly were caused by non-typhoidal *Salmonella*, leading to a loss of $400 million in direct medical costs [9]. Salmonellosis is a leading foodborne pathogen in Egypt; nevertheless, there was no national surveillance with reliable statistics on its health and economic load [10].

The pathogenicity of *Salmonella* species is based on many virulence genes that could cause severe infections, such as the invasion A (*invA*) gene, enterotoxin gene (*Stn*), and *Salmonella* plasmid virulence C protein gene (*spvC*) genes [6]. Many virulence-determinant genes are grouped in specific genomic regions known as *Salmonella* Pathogenicity Islands (SPIs), gained via genetic transfer across bacterial pathogens [11]. *Salmonella* pathogenicity islands (SPI-1 to SPI-5) are located at the large chromosomal DNA region and help in the invasion of the genus to host epithelial cells. SPI-1 and SPI-2 have many genes encoding type III secretion systems (TTSSs), which is a needle-like device that helps *Salmonella* to inject its effectors across the intestinal epithelial cell membrane into the cytoplasm, which permits *Salmonella* to rearrangement of the actin cytoskeleton in host epithelial cell, leading to ruffling (outward extension) of the epithelial cell membrane to engulf the bacteria [6]. The *invA* gene (SPI-1 gene) is the *Salmonella* marker gene that allows *Salmonella* to invade host epithelial cells [12]. The enterotoxin *stn* gene has biological activity like that of the cholera toxin (CT) and encodes a protein that causes gastroenteritis with various symptoms including nausea, vomiting, abdominal pain, fever, and diarrhea [12, 13]. The *spvC* gene has a significant role in the survival of *Salmonella* in host cells and reaction with the host defense mechanism and reduction of cytokine production, additionally, it helps systemic invasion of *Salmonella* in the host cells and could be used as a standard for detecting the pathogenicity of *Salmonella* isolates [14].

The emergence of multidrug-resistant bacteria (MDR) against the commonly used antimicrobials is alarming as it could reduce the therapeutic options for treating complicated *Salmonella* infection cases [7]. Annually, more than 2.8 million people in the United States have been infected by antibiotic-resistant bacteria [9]. *Salmonella* strains isolated from chicken samples exhibit a variety of antibiotic resistance profiles. The MDR bacteria harboring antimicrobial resistance genes can be transmitted through food of animal origin to humans, especially chicken and its giblets [15]. In recent years, *Salmonella* isolates have demonstrated high levels of resistance to the most clinically important antimicrobials, such as cephalosporins and fluoroquinolones, leading to many morbidity and mortality cases globally [16].

The β-lactams antibiotics have a top place in the antibacterial armamentarium and the widespread emergence of multidrug-resistant bacteria carrying beta-lactamase genes among foods of animal origin is considered a worrisome threat to public health. Beta-lactamases are bacterial enzymes that hydrolyze the β-lactam ring in β-lactam antibiotics. The most common β-lactamase resistance genes are *bla*_OXA_, *bla*_CTX_, *bla*_SHV_, and *bla*_TEM_ [17]. The most predominant β-lactamases in multi-drug-resistant *Salmonella* isolates are the CTX-M family, followed by TEM and SHV [18]. Extended-spectrum β-lactamases (ESBLs) confer resistance to third-generation cephalosporins (cefotaxime, ceftriaxone, and ceftazidime) [19].

The emergence of MDR *Salmonella* strains, particularly resistant ones to the most critically important antimicrobial classes such as polymyxin, fluoroquinolones, and third- and fourth-generation cephalosporins, is worrisome. The current study, therefore, intended to determine the prevalence, virulence genes (*invA*, *stn*, and *spvC* genes), antimicrobial resistance profiles, and β-lactamase resistance genes (*bla*_OXA_, *bla*_CTX-M1_, *bla*_SHV_, and *bla*_TEM_) of *Salmonella* strains isolated from native chicken carcasses in Egypt marketed in Mansoura, Egypt, as well as highlight the hazard of isolated MDR-, colistin-, cefepime-, and levofloxacin-resistant *Salmonella* enterica serovars to public health.

## Materials and methods

### Collection and preparation of samples

A total of 150 freshly dressed chicken samples were purchased from various poultry shops with various sanitation levels in Mansoura city, Egypt during the period from July 2022 to November 2022. Whole chicken carcasses were separately packed in sterile polyethylene bags, held at 4 °C in an insulated ice box, and transported within an hour to the Laboratory of Food Hygiene, Safety, and Technology Department, Faculty of Veterinary Medicine, Mansoura University, Egypt, wherein the bacteriological analyses for *Salmonella* were performed immediately*.*

### Isolation of *Salmonella*

The preparation of chicken samples, isolation, and identification of *Salmonella* was done according to the methodology recommended by the Food Safety and Inspection Service of the United States Department of Agriculture [20]. Each chicken carcass was weighed (ranging from 1.0 kg to 1.8 kg), placed in a Whirl–Pak bag, and then 400 ml of sterile buffered peptone water (BPW; CM0509B; Oxoid Ltd., Basingstoke, UK) was added. The carcass was rinsed manually for 10 min to ensure that BPW was in contact with the external and internal surfaces of the chicken carcass, and then the chicken rinsate was mixed with buffered peptone water and incubated at 37 °C for 20–24 h.

*Salmonella* isolation was performed according to the International Organization for Standardization (ISO) [21]. From the incubation, an enrichment was done by inoculating 0.1 ml of cultured buffered peptone water into 10 ml of Rappaport–Vassiliadis broth (RV; CM0669; Oxoid Ltd., Basingstoke, UK) then incubated at 42 °C for 20–24 h. After incubation, a loopful from each enriched broth was streaked onto the selective solid media; xylose-lysine-desoxycholate (XLD) agar (Oxoid, CM0469; Oxoid Ltd., Basingstoke, UK) then the inoculated plates were incubated at 37 °C for 24 h. All typical presumptive *Salmonella* colonies (pink with or without black center) on XLD agar were picked up and cultured onto nutrient agar plates (CM0003; Oxoid Ltd., Basingstoke, UK) and incubated at 37 °C for 24 h. Presumptive *Salmonella* colonies were exposed to additional confirmation by biochemical, molecular, and serological identifications. Biochemical tests conducted were triple sugar iron (TSI; Oxoid Ltd., Basingstoke, UK) test, urease test (CM0053B, urease Agar Base (Christensen), Oxoid Ltd., Basingstoke, UK), indole production (SIM medium, CM0435, Oxoid Ltd., Basingstoke, UK), methyl-red (MR) test (MRVP medium, CM0043, Oxoid Ltd., Basingstoke, UK), Simmons citrate (Titan media, India) test, and Voges-Proskauer (VP) test (MRVP MEDIUM, CM0043, Oxoid Ltd., Basingstoke, UK). Isolates confirmed biochemically to be *Salmonella* were serologically identified.

### Serological identification of *Salmonella* isolates

PCR-verified *Salmonella* isolates were classified into serovars according to the Kauffmann–White scheme by slide agglutination test depending on monovalent and polyvalent O and H antisera (Denka-Seiken, Tokyo, Japan) [22].

### Molecular detection of virulence genes in *Salmonella* isolates

The biochemically verified *Salmonella* isolates were further confirmed by applying the polymerase chain reaction (PCR) targeting the *invA*, *stn*, and *spvC* genes. According to the manufacturer's prescript, the genomic DNA of suspected *Salmonella* isolates was extracted using QIAamp® genomic DNA extraction kits (QIAGEN, Germantown, MD, USA).

Detection of the invasion gene (*invA*) was performed using the forward (5′-ACAGTGCTCGTTTACGACCTGAAT-3′) and the reverse (5′-AGACGACTGGTACTGATCGATAAT-3′) primer sequence sets, which yield an amplified band size of 244 bp [23]. Detection of the enterotoxin gene (*stn*) was performed with the forward (5′-CTTAATCGCGCCGCCATGCTGTT-3′) and the reverse (5′- CATGAACTGGCGCAGGTGAT-3′) primer sequence which produces an amplified DNA size of 480 bp [24]. The detection of the *Salmonella* plasmid virulence gene (*spvC*) was performed with primer sequence sets (forward: 5′-ACCAGAGACATTGCCTTCC-3′; and reverse: 5′-TTCTGATCGCCGCTATTCG -3′) which yield an amplified DNA size of 467 bp [25].

The PCR amplification of *invA*, *stn*, and *spvC* genes was applied using a SimpliAmp thermal cycler (Thermo Fisher Scientific Inc, UK). The protocol of PCR cycling for the three genes detected was done as an initial denaturation at 95 °C for 2 min, followed by 35 cycles of denaturation at 95 °C for 10 s, annealing at 60 °C for 30 s, and extension at 72 °C for 30 s, followed by a final extension at 72 °C for 5 min. Ten microliters of each amplified PCR product were electrophoresed in 1.5% agarose gel (Puregene™, India) for 50 min at 95 V and then visualized under an ultraviolet transilluminator (acculab, Montréal, Québec, Canada). A 100-bp DNA ladder (Solarbio; Beijing Solarbio Science & Technology Co., Ltd., China) was used as a marker for PCR products.

### Antibiotic susceptibility testing for *Salmonella* isolates

Antibiogram profiles of 129 molecularly-verified *Salmonella* isolates against sixteen antimicrobials related to eleven antibiotic classes were done using the disk-diffusion method on the Mueller–Hinton agar (MH; CM0337; Oxoid Ltd., Basingstoke, UK) for all the antibiotics tested except the polymyxins class (Colistin), where the minimum inhibition concentration (MIC) was applied and determined by broth microdilution technique according to Clinical and Laboratory Standards Institute guidelines [26]. *Salmonella* isolates with colistin MICs ≥ 4 μg/ml were interpreted as resistant.

The isolates were tested against various classes of antibiotics which included polymyxins class (Colistin), Carbapenems (Meropenem, MEM—10 μg), Sulfonamides (Trimethoprim/Sulphamethoxazole, SXT—25 μg), Quinolones (Nalidixic acid, NA—30 μg; Levofloxacin, LEV—5μg; Ciprofloxacin, CIP—5 μg), Tetracyclines (Tetracycline, TE—30 μg), Aminoglycosides (Gentamicin, CN—10 μg), Cephalosporins (Cephalothin, KF—30 μg; Cefaclor, CEC—30 μg; Cefotaxime, CTX—30 μg; Cefepime, FEP—30 μg), Macrolides (Azithromycin, AZM—15 μg), Phosphonic antibiotics (Fosfomycin, FOS—50 μg), Extended-spectrum beta-lactamases (Ceftazidime/Clavulanic acid, CAZ/CLA—10/30 μg), Glycopeptides (Vancomycin, VA—30 μg). All antibiotic discs were purchased from Oxoid (Oxoid Ltd., Basingstoke, UK).

According to the antimicrobial resistance profiles, *Salmonella* isolates were classified into multidrug-resistant (MDR) if they showed resistance to at least one antimicrobial agent in three or more antimicrobial classes, extensively drug-resistant (XDR) when they were resistant to all tested antimicrobial classes except one or two antimicrobial classes, while considered pan drug-resistant (PDR) when they showed resistance to all tested antimicrobials in all antimicrobial classes [27]. The MAR “multiple antibiotic resistance” index was calculated for all *Salmonella* isolates as the ratio of the number of antimicrobials to which an isolate was resistant to the total number of antimicrobials tested [28]. MAR index > 0.2 implies high-risk contamination and the misuse of antibiotics.

### Detection of β-lactamase resistance genes

Cefotaxime-resistant *Salmonella* isolates (n = 82) were tested using multiplex polymerase chain reaction targeting the β-lactamase resistance genes. The adopted primer set sequences and DNA amplification protocol were previously described by Perez et al. [29] for *bla*_*OXA*_, *bla*_*CTX-M1*_, and *bla*_*TEM*_ genes and Ogutu et al. [30] for the *bla*_*SHV*_ gene. Primers used in this study were constructed to yield 564, 655, 713, and 800 bp for *bla*_*OXA*_, *bla*_*CTX-M1*_, *bla*_*SHV*_, and *bla*_*TEM*_, respectively. The protocol of PCR cycling for these genes was done as an initial denaturation at 94 °C for 10 min, followed by 35 cycles of denaturation at 94 °C for 30 s, annealing at 61 °C for 35 s, and extension at 72 °C for 1 min, followed by a final extension at 72 °C for 8 min. Ten microliters of each amplified PCR product were electrophoresed in 1.5% agarose gel for 90 min at 80 V and then visualized under an ultraviolet transilluminator. A 100-bp DNA ladder was used as a marker for PCR products.

### Statistical analysis

Data analysis was performed using the SPSS program (SPSS Inc., Chicago, IL; v 21). The distribution of virulence genes and resistance rates of *Salmonella* serovars isolated against different antimicrobial agents tested was determined using the chi-square (χ2) test.

## Results

One hundred and fifty freshly dressed native chicken carcasses were collected from different poultry shops to isolate *Salmonella* species using standard bacteriological techniques, comprising pre-enrichment in BPW, selective enrichment in RVS broth, and cultivating on the surface of XLD agar. All presumptive isolates were subjected to biochemical tests, serological identification, and PCR targeting the *invA* gene. *Salmonella isolates* were examined for the existence of two selected virulence genes (*stn* and *spvC*) and antimicrobial susceptibility testing by Kirby–Bauer disc diffusion method for all (n = 16) antimicrobial agents tested, except for colistin, where the MIC was determined by broth microdilution technique. Additionally, cefotaxime-resistant *Salmonella* isolates (n = 82) were tested using multiplex PCR targeting the β-lactamase resistance genes, including *bla*_OXA_, *bla*_CTX-M1_, *bla*_SHV_, and *bla*_TEM_ genes.

### Phenotypic characteristics of the recovered *Salmonella* isolates

Conventional cultural and morphological characteristics revealed 357 (51/150; 43%) suspected *Salmonella* isolates. The morphological characters of presumptive *Salmonella* colonies on XLD were pink colonies with or without black centers. Based on the biochemical tests, *Salmonella* isolates are Voges-Proskauer, urease, and indole negative, and positive TSI, methyl red, and Citrate utilization tests.

### Prevalence of *Salmonella* spp. in freshly dressed chicken carcasses

Of the 357 presumptive *Salmonella* isolates determined based on conventional cultural and biochemical identification methods, only 129 isolates from 27 native chicken carcasses were confirmed as *Salmonella* depending on molecular identification of the *invA Salmonella* marker gene with an overall prevalence of 18% (27/150). *Salmonella* prevalence rate decreased after molecular confirmation compared to conventional cultural and biochemical identification methods, indicating the accuracy and reliability of molecular techniques.

### Serotypes of *Salmonella* isolates recovered from chicken carcasses

**Fig. 1:**
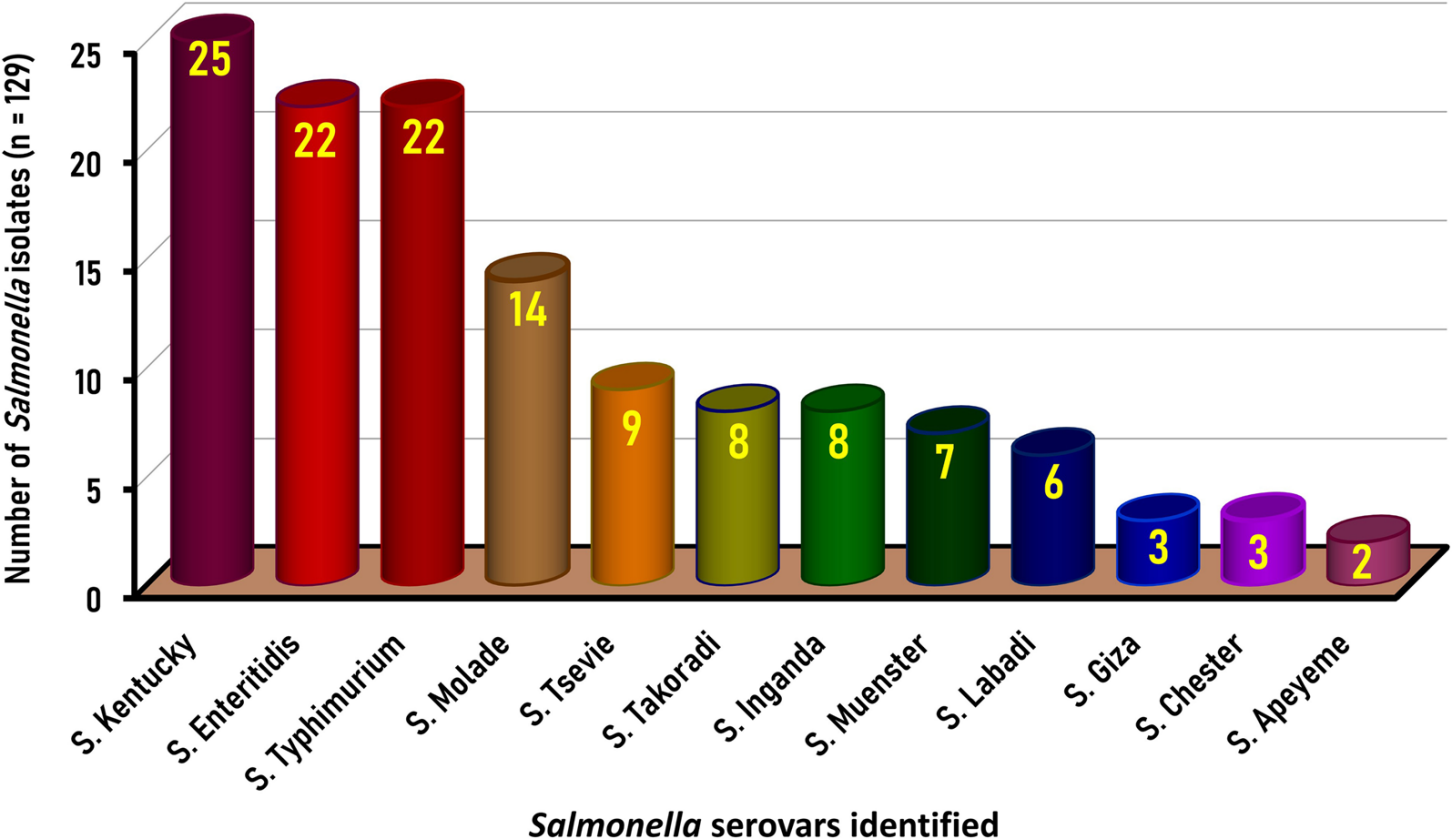
Serological identification of the 129 *Salmonella enterica* Serovars isolates recovered from native Egyptian chicken carcasses

The molecularly confirmed *Salmonella* isolates (n = 129) were serologically identified into 12 different *Salmonella* serotypes. *Salmonella* Kentucky, *S*. Enteritidis, *S*. Typhimurium, and *S*. Molade were the most prevalent serotypes with an incidence of 19.4% (25/129), 17.1% (22/129), 17.1% (22/129), and 10.9% (14/129), respectively. Moreover, *S*. Tsevie, *S*. Takoradi, *S*. Inganda, *S*. Muenster, and *S*. Labadi were detected with an incidence of 6.9% (9/129), 6.2% (8/129), 6.2% (8/129), 5.4% (7/129), and 4.6% (6/129), respectively. While the less common serotypes were *S*. Giza (2.3%, 3/129), *S*. Chester (2.3%, 3/129), and *S*. Apeyeme (1.6%, 2/129) (Fig. 1).

### Prevalence, and distribution of virulence genes among *Salmonella* isolated from chicken carcasses

**Fig. 2:**
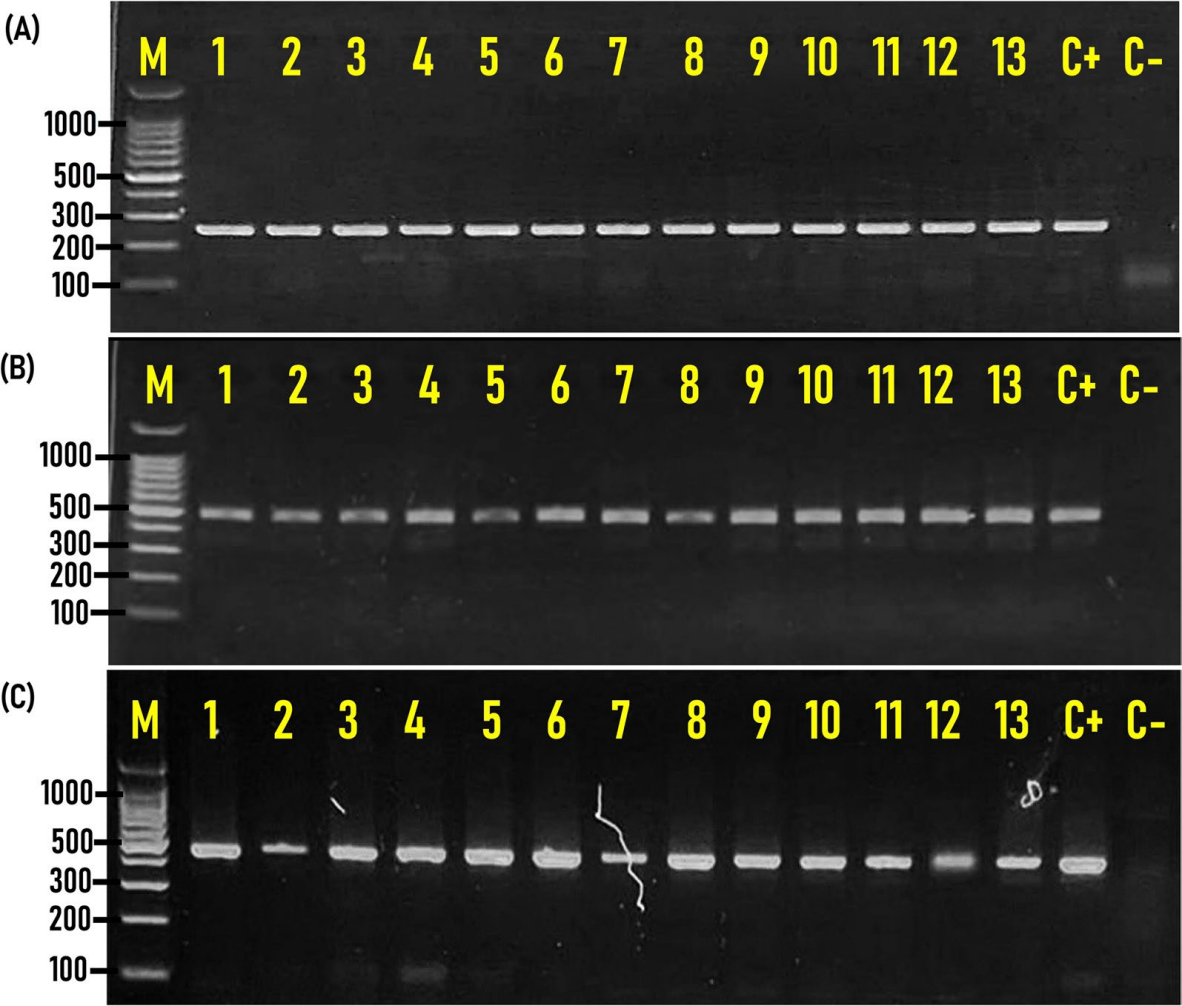
Representative agarose gel electrophoresis for PCR assay of the virulence genes detected in *Salmonella* species. **A** *invA* (244 bp); **B** *stn* (480 bp); **C** *spvC* (467 bp) in *Salmonella* isolates recovered from native Egyptian chicken carcasses. M; DNA marker (100-bp gene ladder). C+; Control positive, C–Control negative. Ten microliters of the PCR product were separated by electrophoresis on 1.5% agarose gel and visualized under UV light

Only 129 of the 356 isolates detected by conventional cultural and biochemical identification methods were molecularly confirmed as *Salmonella* via PCR amplification of the 244-bp DNA fragment of the *invA* marker gene specific for *Salmonella* species. The other virulence genes detected were *stn*, and *spvC*, which were amplified at a molecular size of 480, and 467 bp, respectively (Fig. 2). All tested *Salmonella* isolates were positive for both *invA* and *stn* genes, while only 31.8% (41/129) of isolates examined were positive for the *spvC* gene (Fig. 2).

### Antimicrobial resistance of *Salmonella* enterica serovars isolates (n = 129) recovered from native Egyptian chicken carcasses

The *Salmonella* isolates in the current study showed a high resistance rate of 92.3% (119/129), 82.9% (107/129), 82.2% (106/129), 82.2% (106/129), and 63.6% (82/129) against vancomycin, nalidixic acid, cefepime, colistin, and cefotaxime, respectively (Table 1), while they exhibited a mediate resistance rate of 51.9% (67/129), 50.4% (65/129), 50.4% (65/129), 48.8% (63/129), 47.3(61/129), and 42.6% (55/129) against ceftazidime/clavulanic acid, levofloxacin, tetracycline, ciprofloxacin, cefaclor, and cephalothin, respectively (Table 1). On the other hand, *Salmonella* isolates showed lower resistance rates of 32. 6% (42/129), 31% (40/129), 28.7% (37/129), 11.6% (15/129), and 10.1% (13/129) towards gentamicin, sulfamethoxazole/trimethoprim, Fosfomycin, azithromycin, and meropenem, respectively (Table 1).

**Table 1:** Antimicrobial susceptibility of *Salmonella enterica* serovars isolates (n = 129) recovered from native Egyptian chicken carcasses

No	Antimicrobial agent (acronym)	Sensitive		Intermediate		Resistant	
		No	%	No	%	No	%
1	Vancomycin (VA)	10	7.7	–	–	119	92.3
2	Nalidixic acid (NA)	22	17.1	–	–	107	82.9
3	Cefepime (FEP)	–	–	23	17.8	106	82.2
4	Colistin (CT)	5	3.9	18	13.9	106	82.2
5	Cefotaxime (CTX)	12	9.3	35	27.1	82	63.6
6	Ceftazidime/Clavulanic acid (CAZ/CLA)	7	5.4	55	42.6	67	51.9
7	Levofloxacin (LEV)	7	5.4	57	44.2	65	50.4
8	Tetracycline (TE)	49	37.9	15	11.6	65	50.4
9	Ciprofloxacin (CIP)	13	10.1	53	41.1	63	48.8
10	Cefaclor (CEC)	23	17.8	45	34.9	61	47.3
11	Cephalothin (KF)	62	48.1	12	9.3	55	42.6
12	Gentamicin (CN)	76	58.9	11	8.5	42	32.6
13	Sulfamethoxazole/Trimethoprim (SXT)	85	65.9	4	3.1	40	31
14	Fosfomycin (FOS)	61	47.3	31	24	37	28.7
15	Azithromycin (AZM)	114	88.4	–	–	15	11.6
16	Meropenem (MEM)	109	84.5	7	5.4	13	10.1

### Classification of *Salmonella* isolates based on their antibiotic resistance profile and their multiple antibiotic resistance (MAR) index

The antimicrobial resistance profile of all *Salmonella* isolates (n = 129) examined toward 16 antimicrobial agents revealed 48 different patterns and 121 (93.8%) of the 129 *Salmonella*-verified isolates were resistant to at least 3 antibiotics (Table 2). Furthermore, 3.9% (5/129), 14.7% (19/129), 75.2% (97/129), and 6.2% (8/129) of *Salmonella* isolates tested were categorized according to their antibiotic resistance phenotype into pan-drug-resistant, extensively drug-resistant, multidrug-resistant, and low drug-resistant, respectively (Table 2). Amazingly, five isolates (3.9%, 5/129) comprised 4 *Salmonella* Enteritidis isolates and 1 *Salmonella* Typhimurium isolate revealed resistance to all antimicrobial agents tested with a MAR index equal to 1 (Table 3).

**Table 2:** Antimicrobial resistance profile and MAR indexes of *Salmonella* serovars isolates (n = 129) from native Egyptian chicken carcasses

Antimicrobial resistance patterns	Number and (%) of isolates	MAR index	Resistance profile	Number and (%) for each profile
VA, FEP, CT, NA, CTX, LEV, CAZ/CLA, TE, CIP, CEC, FOS, KF, CN, SXT, AZM, MEM	5 (3.88%)	1	Pan-drug-resistant	5 (3.9%)
VA, FEP, CT, NA, CTX, LEV, CAZ/CLA, TE, CIP, CEC, FOS, KF, CN, SXT, AZM	2 (1.55%)	0.9375	Extensively drug-resistant	19 (14.7%)
VA, FEP, CT, NA, CTX, LEV, CAZ/CLA, TE, CIP, CEC, FOS, KF, SXT, MEM	3 (2.33%)	0.875		
VA, FEP, CT, NA, CTX, LEV, CAZ/CLA, TE, CIP, CEC, FOS, KF, CN, MEM	2 (1.55%)	0.875		
VA, FEP, CT, NA, CTX, LEV, CAZ/CLA, TE, CIP, CEC, KF, CN, SXT, AZM	3 (2.33%)	0.875		
VA, FEP, CT, NA, CTX, LEV, CAZ/CLA, TE, CIP, CEC, FOS, KF, CN, SXT	9 (6.98%)	0.875		
VA, FEP, CT, NA, CTX, LEV, CAZ/CLA, TE, CIP, CEC, FOS, KF, SXT	3 (2.33%)	0.8125	Multidrug-resistant	97 (75.2%)
VA, FEP, CT, NA, CTX, LEV, CAZ/CLA, TE, CIP, CEC, KF, CN, SXT	7 (5.43%)	0.8125		
VA, FEP, NA, CTX, LEV, CAZ/CLA, CIP, CEC, KF, CN, SXT, AZM	1 (0.78%)	0.750		
VA, FEP, CT, NA, CTX, LEV, CAZ/CLA, TE, CIP, CEC, KF, CN	4 (3.1%)	0.750		
VA, FEP, CT, NA, CTX, LEV, CAZ/CLA, TE, CIP, KF, CN, SXT	1 (0.78%)	0.750		
VA, FEP, CT, NA, CTX, LEV, TE, CIP, CEC, CN, SXT, AZM	1 (0.78%)	0.750		
VA, FEP, CT, NA, CTX, LEV, CAZ/CLA, TE, CIP, CEC, KF	7 (5.43)	0.6875		
VA, FEP, CT, NA, CTX, LEV, TE, CIP, CEC, CN, SXT	1 (0.78%)	0.6875		
VA, FEP, CT, NA, LEV, CAZ/CLA, TE, CIP, CEC, SXT	1 (0.78%)	0.625		
VA, FEP, NA, CTX, CAZ/CLA, TE, CIP, CN, SXT	1 (0.78%)	0.5625		
VA, FEP, NA, CTX, LEV, TE, CIP, SXT, AZM	1 (0.78%)	0.5625		
VA, FEP, CT, NA, CTX, CAZ/CLA, TE, CEC	2 (1.55%)	0.500		
VA, CT, LEV, CAZ/CLA, TE, CIP, CEC, KF	2 (1.55%)	0.500		
VA, CT, NA, CTX, LEV, TE, CIP, KF	2 (1.55%)	0.500		
VA, NA, LEV, CAZ/CLA, TE, CEC, SXT	1 (0.78%)	0.4375		
VA, FEP, CT, NA, CAZ/CLA, FOS, CN	1 (0.78%)	0.4375		
VA, FEP, CT, NA, CAZ/CLA, TE, CEC	3 (2.33%)	0.4375		
VA, NA, LEV, CEC, KF, CN, AZM	2 (1.55%)	0.4375		
FEP, CT, CTX, LEV, TE, CN, SXT	1 (0.78%)	0.4375		
VA, FEP, CT, NA, CTX, CIP, CEC	2 (1.55%)	0.4375		
VA, FEP, CT, NA, LEV, TE, CIP	2 (1.55%)	0.4375		
VA, FEP, CT, NA, CAZ/CLA, FOS	2 (1.55%)	0.375		
VA, FEP, CT, NA, LEV, TE	1 (0.78%)	0.375		
VA, FEP, CT, NA, CTX, FOS	5 (3.88%)	0.375		
FEP, CT, NA, CAZ/CLA, FOS	2 (1.55%)	0.3125		
VA, CT, NA, CTX, CAZ/CLA	3 (2.33%)	0.3125		
VA, FEP, CT, NA, CAZ/CLA	2 (1.55%)	0.3125		
VA, FEP, CT, CTX, FOS	1 (0.78%)	0.3125		
VA, FEP, CT, NA, CTX	5 (3.88%)	0.3125		
VA, FEP, NA, CTX	4 (3.1%)	0.250		
VA, FEP, LEV, CIP	3 (2.33%)	0.250		
VA, FEP, CT, CTX	2 (1.55%)	0.250		
VA, CT, CTX, KF	2 (1.55%)	0.250		
VA, FEP, CT, NA	4 (3.1%)	0.250		
CT, NA, CTX	2 (1.55%)	0.1875		
VA, FEP, NA	4 (3.1%)	0.1875		
VA, FEP, CT	8 (6.2%)	0.1875		
VA, CT, NA	1 (0.78%)	0.1875		
NA, MEM	3 (2.33%)	0.125	Low drug-resistant	8 (6.2%)
VA, FOS	2 (1.55%)	0.125		
CT, NA	2 (1.55%)	0.125		
VA	1 (0.78%)	0.0625		

VA, Vancomycin; FEP, Cefepime; CT, Colistin; NA, Nalidixic acid; CTX, Cefotaxime; LEV, Levofloxacin; TE, Tetracycline; CAZ/CLA, Ceftazidime/Clavulanic acid; CIP, Ciprofloxacin; CEC, Cefaclor; KF Cephalothin; CN, Gentamicin; SXT, Sulfamethoxazole/Trimethoprim; FOS, Fosfomycin; AZM, Azithromycin; MEM, Meropenem

**Table 3:** Classification of *Salmonella enterica* server isolates (n = 129) according to their antimicrobial resistance profile against the 16 antimicrobial agents tested

Serovars	Number of isolates	Antimicrobial resistance pattern	Antimicrobial resistance classes	MAR Index	Classification of Strains	
					Type of resistance	No. and %
*Salmonella enterica* subsp. enterica serovar Kentucky (n = 25)	4	VA, FEP, CT, NA, CTX, LEV, CAZ/CLA, TE, CIP, CEC, KF, CN, SXT	Glycopeptide, cephalosporin, Polymyxin, Quinolone, Fluoroquinolone, ESBL, tetracyclines, aminoglycoside, Sulfonamides	0.8125	Multidrug-resistant	22 (88%)
	3	VA, FEP, CT, NA, CTX, LEV, CAZ/CLA, TE, CIP, CEC, FOS, KF, SXT	Glycopeptide, cephalosporin, Polymyxin, Quinolone, Fluoroquinolone, ESBL, tetracyclines, Phosphonic, Sulfonamides	0.8125		
	3	VA, FEP, CT, NA, CTX, LEV, CAZ/CLA, TE, CIP, CEC, KF	Glycopeptide, cephalosporin, Polymyxin, Quinolone, Fluoroquinolone, ESBL, tetracyclines	0.6875		
	2	VA, CT, LEV, CAZ/CLA, TE, CIP, CEC, KF	Glycopeptide, cephalosporin, Polymyxin, Fluoroquinolone, ESBL, tetracyclines	0.500		
	2	VA, FEP, CT, NA, CTX, FOS	Glycopeptide, cephalosporin, Polymyxin, Quinolone, Phosphonic	0.375		
	4	VA, FEP, NA	Glycopeptide, cephalosporin, Quinolone	0.1875		
	2	VA, FEP, CT	Glycopeptide, cephalosporin, Polymyxin	0.1875		
	2	CT, NA, CTX	Cephalosporin, Polymyxin, Quinolone	0.1875		
	3	NA, MEM	Quinolone, carbapenem	0.125	Low drug-resistant	3 (12%)
Average MAR Index for *Salmonella Kentucky *= 0.455						
*Salmonella enterica* subsp. enterica serovar Enteritidis (n = 22)	4	VA, FEP, CT, NA, CTX, LEV, CAZ/CLA, TE, CIP, CEC, FOS, KF, CN, SXT, AZM, MEM	Glycopeptide, cephalosporin, Polymyxin, Quinolone, Fluoroquinolone, ESBL, tetracyclines, Phosphonic, aminoglycoside, Sulfonamides, Macrolide, carbapenem	1.000	Pan-drug-resistant	4 (18.18%)
	3	VA, FEP, CT, NA, CTX, LEV, CAZ/CLA, TE, CIP, CEC, FOS, KF, CN, MEM	Glycopeptide, cephalosporin, Polymyxin, Quinolone, Fluoroquinolone, ESBL, tetracyclines, Phosphonic, aminoglycoside, carbapenem	0.875	Extensively drug-resistant	5 (22.73%)
	2	VA, FEP, CT, NA, CTX, LEV, CAZ/CLA, TE, CIP, CEC, FOS, KF, SXT, MEM	Glycopeptide, cephalosporin, Polymyxin, Quinolone, Fluoroquinolone, ESBL, tetracyclines, Phosphonic, Sulfonamides, carbapenem	0.875		
	2	VA, FEP, CT, NA, CTX, CAZ/CLA, TE, CEC	Glycopeptide, cephalosporin, Polymyxin, Quinolone, ESBL, tetracyclines, Phosphonic, Sulfonamides, carbapenem	0.500	Multidrug-resistant	13 (59.09%)
	3	VA, FEP, CT, NA, CTX	Glycopeptide, cephalosporin, Polymyxin, Quinolone	0.3125		
	2	VA, FEP, NA, CTX	Glycopeptide, cephalosporin, Quinolone	0.250		
	3	VA, FEP, LEV, CIP	Glycopeptide, cephalosporin, Fluoroquinolone	0.250		
	3	VA, FEP, CT	Glycopeptide, cephalosporin, Polymyxin	0.1875		
Average MAR Index for *Salmonella enteritidis* = 0.551						
*Salmonella enterica* subsp. enterica serovar Typhimurium (n = 22)	1	VA, FEP, CT, NA, CTX, LEV, CAZ/CLA, TE, CIP, CEC, FOS, KF, CN, SXT, AZM, MEM	Glycopeptide, cephalosporin, Polymyxin, Quinolone, Fluoroquinolone, ESBL, tetracyclines, Phosphonic, aminoglycoside, Sulfonamides, Macrolide, carbapenem	1.000	Pan-drug-resistant	1 (4.54%)
	3	VA, FEP, CT, NA, CTX, LEV, CAZ/CLA, TE, CIP, CEC, FOS, KF, CN, SXT	Glycopeptide, cephalosporin, Polymyxin, Quinolone, Fluoroquinolone, ESBL, tetracyclines, Phosphonic, aminoglycoside, Sulfonamides	0.875	Extensively drug-resistant	3 (13.64%)
	4	VA, FEP, CT, NA, CTX, LEV, CAZ/CLA, TE, CIP, CEC, KF, CN	Glycopeptide, cephalosporin, Polymyxin, Quinolone, Fluoroquinolone, ESBL, tetracyclines, aminoglycoside	0.750	Multidrug-resistant	18 (81.82%)
	4	VA, FEP, CT, NA, CTX, LEV, CAZ/CLA, TE, CIP, CEC, KF	Glycopeptide, cephalosporin, Polymyxin, Quinolone, Fluoroquinolone, ESBL, tetracyclines	0.6875		
	2	VA, CT, NA, CTX, LEV, TE, CIP, KF	Glycopeptide, cephalosporin, Polymyxin, Quinolone, Fluoroquinolone, ESBL, tetracyclines	0.500		
	3	VA, CT, NA, CTX, CAZ/CLA	Glycopeptide, cephalosporin, Polymyxin, Quinolone, ESBL	0.3125		
	2	FEP, CT, NA, CAZ/CLA, FOS	Cephalosporin, Polymyxin, Quinolone, ESBL, Phosphonic	0.3125		
	1	VA, FEP, CT, CTX	Glycopeptide, cephalosporin, Polymyxin	0.250		
	2	VA, FEP, CT, NA	Glycopeptide, cephalosporin, Polymyxin, Quinolone	0.250		
Average MAR Index for *Salmonella typhimurium *= 0.577						
*Salmonella enterica* subsp. enterica serovar Molade (n = 14)	3	VA, FEP, CT, NA, CTX, LEV, CAZ/CLA, TE, CIP, CEC, KF, CN, SXT, AZM	Glycopeptide, cephalosporin, Polymyxin, Quinolone, Fluoroquinolone, ESBL, tetracyclines, aminoglycoside, Sulfonamides, Macrolide	0.875	Extensively drug-resistant	3 (21.43%)
	1	VA, FEP, NA, CTX, LEV, CAZ/CLA, CIP, CEC, KF, CN, SXT, AZM	Glycopeptide, cephalosporin, Quinolone, Fluoroquinolone, ESBL, aminoglycoside, Sulfonamides, Macrolide	0.750	Multidrug-resistant	8 (57.14%)
	2	VA, NA, LEV, CEC, KF, CN, AZM	Glycopeptide, cephalosporin, Quinolone, Fluoroquinolone, aminoglycoside, Macrolide	0.4375		
	2	VA, FEP, NA, CTX	Glycopeptide, cephalosporin, Quinolone	0.250		
	3	VA, FEP, CT	Glycopeptide, cephalosporin, Polymyxin	0.1875		
	2	CT, NA	Polymyxin, Quinolone	0.125	Low drug-resistant	3 (21.43%)
	1	VA	Glycopeptide	0.0625		
Average MAR Index for *Salmonella* molade = 0.402						
*Salmonella enterica* subsp. enterica serovar Tsevie (n = 9)	3	VA, FEP, CT, NA, CTX, LEV, CAZ/CLA, TE, CIP, CEC, FOS, KF, CN, SXT	Glycopeptide, cephalosporin, Polymyxin, Quinolone, Fluoroquinolone, ESBL, tetracyclines, Phosphonic, aminoglycoside, Sulfonamides	0.875	Extensively drug-resistant	3 (33.33%)
	1	VA, FEP, CT, NA, LEV, CAZ/CLA, TE, CIP, CEC, SXT	Glycopeptide, cephalosporin, Polymyxin, Quinolone, Fluoroquinolone, ESBL, tetracyclines, Sulfonamides	0.625	Multidrug-resistant	4 (44.45%)
	1	VA, NA, LEV, CAZ/CLA, TE, CEC, SXT	Glycopeptide, cephalosporin, Quinolone, Fluoroquinolone, ESBL, tetracyclines, Sulfonamides	0.4375		
	2	VA, FEP, CT, NA, CTX	Glycopeptide, cephalosporin, Polymyxin, Quinolone	0.3125		
	2	VA, FOS	Glycopeptide, Phosphonic	0.125	Low drug-resistant	2 (22.22%)
Average MAR Index for *Salmonella tsevie *= 0.507						
*Salmonella enterica* subsp. enterica serovar Takoradi (n = 8)	3	VA, FEP, CT, NA, CTX, LEV, CAZ/CLA, TE, CIP, CEC, FOS, KF, CN, SXT	Glycopeptide, cephalosporin, Polymyxin, Quinolone, Fluoroquinolone, ESBL, tetracyclines, Phosphonic, aminoglycoside, Sulfonamides	0.875	Extensively drug-resistant	3 (37.5%)
	1	VA, FEP, CT, NA, CTX, LEV, CAZ/CLA, TE, CIP, KF, CN, SXT	Glycopeptide, cephalosporin, Polymyxin, Quinolone, Fluoroquinolone, ESBL, tetracyclines, aminoglycoside, Sulfonamides	0.750	Multidrug-resistant	5 (62.5%)
	1	VA, FEP, NA, CTX, CAZ/CLA, TE, CIP, CN, SXT	Glycopeptide, cephalosporin, Quinolone, Fluoroquinolone, ESBL, tetracyclines, aminoglycoside, Sulfonamides	0.5625		
	2	VA, FEP, CT, NA, CAZ/CLA	Glycopeptide, cephalosporin, Polymyxin, Quinolone, ESBL	0.3125		
	1	VA, CT, NA	Glycopeptide, Polymyxin, Quinolone	0.1875		
Average MAR Index for *Salmonella* takoradi = 0.594						
*Salmonella enterica* subsp. enterica serovar Inganda (n = 8)	3	VA, FEP, CT, NA, CAZ/CLA, TE, CEC	Glycopeptide, cephalosporin, Polymyxin, Quinolone, ESBL, tetracyclines	0.4375	Multidrug-resistant	8 (100%)
	3	VA, FEP, CT, NA, CTX, FOS	Glycopeptide, cephalosporin, Polymyxin, Quinolone, Phosphonic	0.375		
	2	VA, CT, CTX, KF	Glycopeptide, cephalosporin, Polymyxin	0.250		
Average MAR Index for *Salmonella* inganda = 0.367						
*Salmonella enterica* subsp. enterica serovar Muenster (n = 7)	3	VA, FEP, CT, NA, CTX, LEV, CAZ/CLA, TE, CIP, CEC, KF, CN, SXT	Glycopeptide, cephalosporin, Polymyxin, Quinolone, Fluoroquinolone, ESBL, tetracyclines, aminoglycoside, Sulfonamides	0.8125	Multidrug-resistant	7 (100%)
	1	VA, FEP, CT, NA, CTX, LEV, TE, CIP, CEC, CN, SXT	Glycopeptide, cephalosporin, Polymyxin, Quinolone, Fluoroquinolone, tetracyclines, aminoglycoside, Sulfonamides	0.6875		
	1	FEP, CT, CTX, LEV, TE, CN, SXT	Glycopeptide, cephalosporin, Polymyxin, Quinolone, Fluoroquinolone, tetracyclines, aminoglycoside, Sulfonamides	0.4375		
	2	VA, FEP, CT, NA	Glycopeptide, cephalosporin, Polymyxin, Quinolone	0.250		
Average MAR Index for *Salmonella* muenster = 0.580						
*Salmonella enterica* subsp. enterica serovar Labadi (n = 6)	2	VA, FEP, CT, NA, CTX, LEV, CAZ/CLA, TE, CIP, CEC, FOS, KF, CN, SXT, AZM	Glycopeptide, cephalosporin, Polymyxin, Quinolone, Fluoroquinolone, ESBL, tetracyclines, Phosphonic, aminoglycoside, Sulfonamides, Macrolide	0.9375	Extensively drug-resistant	2 (33.33%)
	1	VA, FEP, CT, NA, CTX, LEV, TE, CIP, CEC, CN, SXT, AZM	Glycopeptide, cephalosporin, Polymyxin, Quinolone, Fluoroquinolone, ESBL, tetracyclines, Phosphonic, aminoglycoside, Sulfonamides, Macrolide	0.750	Multidrug-resistant	4 (66.67%)
	1	VA, FEP, NA, CTX, LEV, TE, CIP, SXT, AZM	Glycopeptide, cephalosporin, Polymyxin, Quinolone, Fluoroquinolone, ESBL, tetracyclines, Phosphonic, aminoglycoside, Sulfonamides, Macrolide	0.5625		
	2	VA, FEP, CT, NA, CTX, CIP, CEC	Glycopeptide, cephalosporin, Polymyxin, Quinolone, Fluoroquinolone	0.4375		
Average MAR Index for *Salmonella* labadi = 0.677						
*Salmonella enterica* subsp. enterica serovar Giza (n = 3)	2	VA, FEP, CT, NA, LEV, TE, CIP	Glycopeptide, cephalosporin, Polymyxin, Quinolone, Fluoroquinolone, tetracyclines	0.4375	Multidrug-resistant	3 (100%)
	1	VA, FEP, CT, NA, LEV, TE	Glycopeptide, cephalosporin, Polymyxin, Quinolone, Fluoroquinolone, tetracyclines	0.375		
Average MAR Index for *Salmonella giza *= 0.417						
*Salmonella enterica* subsp. enterica serovar Chester (n = 3)	1	VA, FEP, CT, NA, CAZ/CLA, FOS, CN	Glycopeptide, cephalosporin, Polymyxin, Quinolone, ESBL, Phosphonic, aminoglycoside	0.4375	Multidrug-resistant	3 (100%)
	2	VA, FEP, CT, NA, CAZ/CLA, FOS	Glycopeptide, cephalosporin, Polymyxin, Quinolone, ESBL, Phosphonic	0.375		
Average MAR Index for *Salmonella* chester = 0.396						
*Salmonella enterica* subsp. enterica serovar Apeyeme (n = 2)	1	VA, FEP, CT, CTX, FOS	Glycopeptide, cephalosporin, Polymyxin, Phosphonic	0.3125	Multidrug-resistant	2 (100%)
	1	VA, FEP, CT, CTX	Glycopeptide, cephalosporin, Polymyxin	0.250		
Average MAR Index for *Salmonella* Apeyeme=0.281						

VA, Vancomycin; FEP, Cefepime; CT, Colistin; NA, Nalidixic acid; CTX, Cefotaxime; LEV, Levofloxacin; TE, Tetracycline; CAZ/CLA, Ceftazidime/Clavulanic acid; CIP, Ciprofloxacin; CEC, Cefaclor; KF Cephalothin; CN, Gentamicin; SXT, Sulfamethoxazole/Trimethoprim; FOS, Fosfomycin; AZM, Azithromycin; MEM, Meropenem

^**^The average multiple antibiotic resistance (MAR) index for 129 isolates tested was 0.505

### Distribution of β-lactamase resistance genes among MDR *Salmonella* isolates

**Fig. 3:**
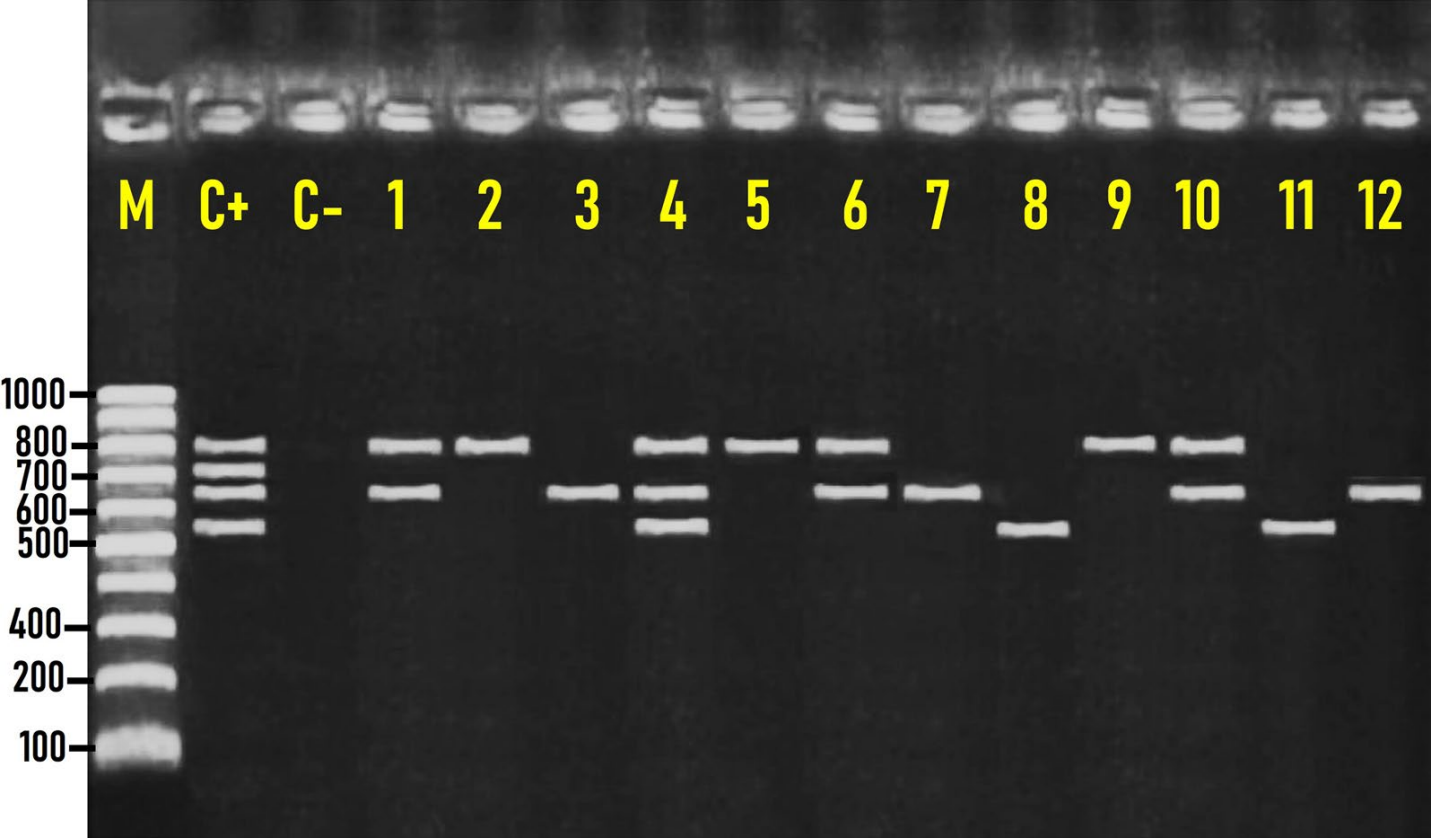
Representative agarose gel electrophoresis for multiplex PCR screening of β-lactamase-resistant genes demonstrated by *bla*_*OXA*_ (564 bp), *bla*_*CTX-M1*_ (655 bp), *bla*_*SHV*_ (713 bp), and *bla*_*TEM*_ (800 bp) detected in *Salmonella* isolates recovered from the native Egyptian chicken carcasses. M; DNA marker (100-bp gene ladder). C+ Control positive, C– Control negative. Lane 1: *S*. Enteritidis (*bla*_*CTX-M1*_- and *bla*_*TEM*_*-*positive); Lane 2: *S*. Labadi (*bla*_*TEM*_*-*positive); Lane 3: *S*. Apeyeme (*bla*_*CTX-M1*_-positive); Lane 4: *S*. Kentucky (*bla*_*OXA*_*-*, *bla*_*CTX-M1*_*-*, and *bla*_*TEM*_-positive); Lane 5: *S*. Enteritidis (*bla*_*TEM*_*-*positive); Lane 6: *S.* Typhimurium (*bla*_*CTX-M1*_- and *bla*_*TEM*_*-*positive); Lane 7: *S.* Typhimurium (*bla*_*CTX-M1*_-positive); Lane 8: *S.* Apeyeme (*bla*_*OXA*_-positive); Lane 9: *S*. Kentucky (*bla*_*TEM*_*-*positive); Lane 10: *S.* Kentucky (*bla*_*CTX-M1*_- and *bla*_*TEM*_*-*positive); Lane 11: *S.* Enteritidis (*bla*_*OXA*_-positive); Lane 12:* S*. Kentucky (*bla*_*CTX-M1*_-positive). Seven microliters of the PCR product were loaded and separated by electrophoresis on 1.5% agarose gel and visualized under UV light

**Fig. 4:**
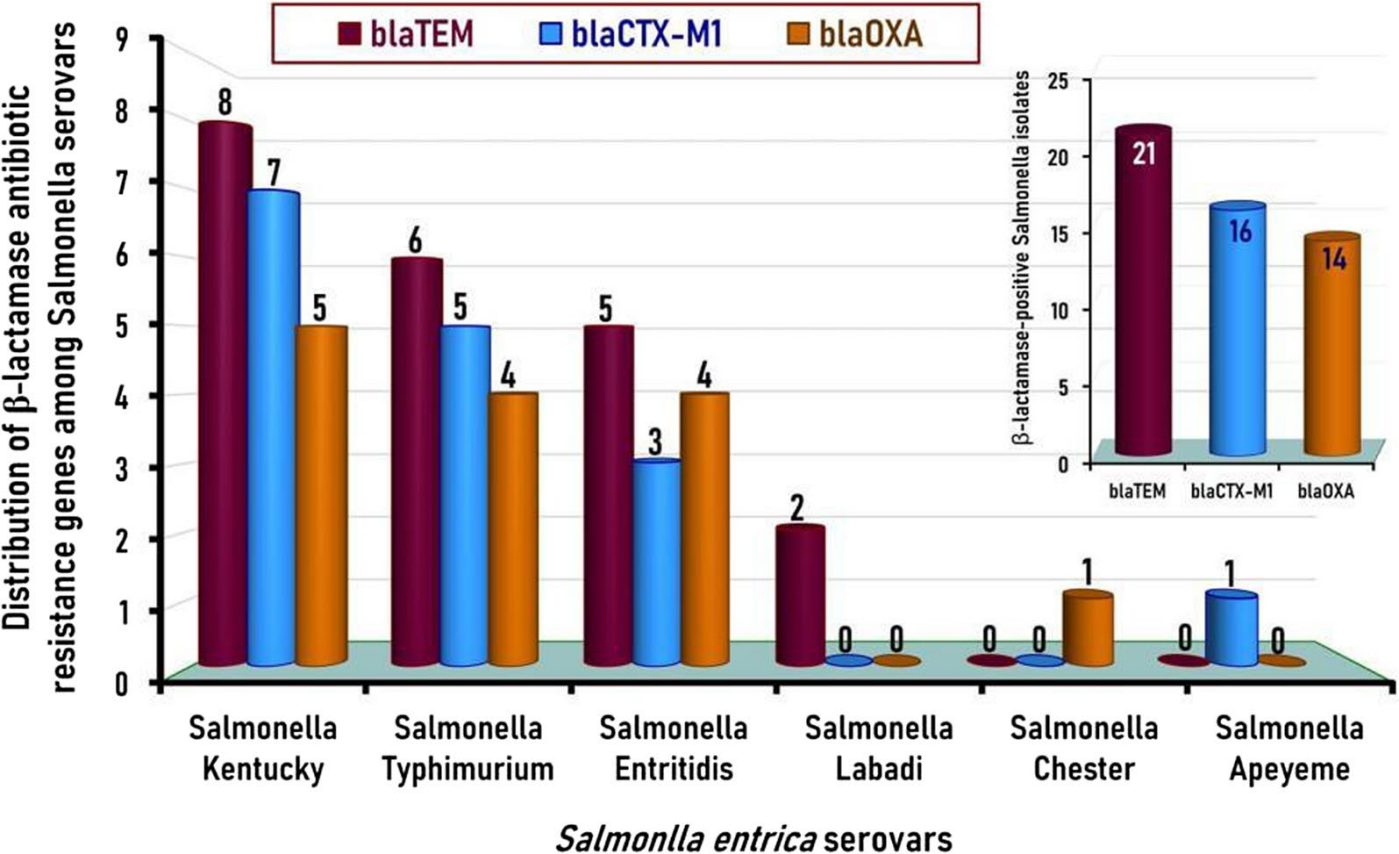
Distribution of the identified β-lactamase resistance genes among the cefotaxime-resistant *Salmonella* isolates (n = 82)

In the present study, β-lactamase-resistance genes encompassing *bla*_OXA_, *bla*_CTX-M1_, *bla*_SHV_, and *bla*_TEM_ were identified in cefotaxime-resistant *Salmonella* isolates at molecular sizes of 564, 655, 713, and 800 bp, respectively (Fig. 3). Thirty-one (37.8%) of the 82 cefotaxime-resistant *Salmonella* isolates tested were β- lactamase producers and had at least one of the *bla*_OXA_, *bla*_CTX-M1_, or *bla*_TEM_ β-lactamase resistance genes (Fig. 4). The distribution of β-lactamase-resistance genes among the positive 31 *Salmonella* serovars indicated that the *bla*_TEM_ was the most predominant β-lactamase resistance gene and was identified in 25.6% (21/82) of the isolates, followed by *bla*_CTX-M1_ and *bla*_OXA_ genes, which were detected in 19.5% (16/82) and 17.1% (14/82) of *Salmonella* isolates tested, respectively (Fig. 4). Conversely, the *bla*_SHV_ gene was not detected in any of the *Salmonella* isolates examined. Interestingly, two isolates comprised one *Salmonella* Kentucky and one *Salmonella* Typhimurium among the 31 β-lactamase producers isolates had the three identified β-lactamase resistance genes: *bla*_OXA_, *bla*_CTX-M1_, and *bla*_TEM_ (Fig. 3 & Table 4).

**Table 4:** Correlation between the phenotypic and genotypic profile of multidrug resistance β-lactamase-producing *Salmonella enterica* serovars (*n *= 31) identified among *Salmonella* isolates retrieved from native Egyptian chicken carcasses

*Salmonella* serovars	No (%) of strains	Antimicrobial resistance	β-Lactamase-resistance genes	Virulence genes
*Salmonella* Kentucky (n = 12)	1	VA, FEP, CT, NA, CTX, LEV, CAZ/CLA, TE, CIP, CEC, KF, CN, SXT	*bla*_TEM_, *bla*_CTX-M1,_ *bla*_OXA_	*invA*, *stn*, *spvC*
	1	VA, FEP, CT, NA, CTX, LEV, CAZ/CLA, TE, CIP, CEC, KF, CN, SXT	*bla*_OXA_	*invA*, *stn*
	1	VA, FEP, CT, NA, CTX, LEV, CAZ/CLA, TE, CIP, CEC, KF, CN, SXT	*bla*_CTX-M1,_ *bla*_OXA_	*invA*, *stn*
	2	VA, FEP, CT, NA, CTX, LEV, CAZ/CLA, TE, CIP, CEC, FOS, KF, SXT	*bla*_TEM,_ *bla*_OXA_	*invA*, *stn*
	1	VA, FEP, CT, NA, CTX, LEV, CAZ/CLA, TE, CIP, CEC, FOS, KF, SXT	*bla*_TEM_, *bla*_CTX-M1_	*invA*, *stn*, *spvC*
	3	VA, FEP, CT, NA, CTX, LEV, CAZ/CLA, TE, CIP, CEC, KF	*bla*_TEM_, *bla*_CTX-M1_	*invA*, *stn*, *spvC*
	1	VA, FEP, CT, NA, CTX, FOS	*bla*_CTX-M1_	*invA*, *stn*, *spvC*
	1	VA, FEP, CT, NA, CTX, FOS	*bla*_OXA_	*invA*, *stn*, *spvC*
	1	CT, NA, CTX	*bla*_TEM_	*invA*, *stn*
*Salmonella* Typhimurium (n = 8)	1	VA, FEP, CT, NA, CTX, LEV, CAZ/CLA, TE, CIP, CEC, FOS, KF, CN, SXT, AZM, MEM	*bla*_TEM_, *bla*_CTX-M1,_ *bla*_OXA_	*invA*, *stn*, *spvC*
	1	VA, FEP, CT, NA, CTX, LEV, CAZ/CLA, TE, CIP, CEC, FOS, KF, CN, SXT	*bla*_CTX-M1_	*invA, stn, spvC*
	2	VA, FEP, CT, NA, CTX, LEV, CAZ/CLA, TE, CIP, CEC, FOS, KF, CN, SXT	*bla*_TEM_, *bla*_CTX-M1_	*invA, stn, spvC*
	1	VA, FEP, CT, NA, CTX, LEV, CAZ/CLA, TE, CIP, CEC, KF, CN	*bla*_TEM_, *bla*_OXA_	*invA*, *stn*
	1	VA, FEP, CT, NA, CTX, LEV, CAZ/CLA, TE, CIP, CEC, KF, CN	*bla*_TEM_, *bla*_OXA_	*invA*, *stn*
	1	VA, FEP, CT, NA, CTX, LEV, CAZ/CLA, TE, CIP, CEC, KF	*bla*_OXA_	*invA*, *stn*
	1	VA, FEP, CT, NA, CTX, LEV, CAZ/CLA, TE, CIP, CEC, KF	*bla*_TEM_, *bla*_CTX-M1_	*invA*, *stn*
*Salmonella* Enteritidis (n = 7)	1	VA, FEP, CT, NA, CTX, LEV, CAZ/CLA, TE, CIP, CEC, FOS, KF, CN, SXT, AZM, MEM	*bla*_TEM_, *bla*_CTX-M1_	*invA*, *stn*, *spvC*
	1	VA, FEP, CT, NA, CTX, LEV, CAZ/CLA, TE, CIP, CEC, FOS, KF, CN, SXT, AZM, MEM	*bla*_TEM_	*invA*, *stn*, *spvC*
	1	VA, FEP, CT, NA, CTX, LEV, CAZ/CLA, TE, CIP, CEC, FOS, KF, CN, SXT, AZM, MEM	*bla*_CTX-M1_, *bla*_OXA_	*invA*, *stn*, *spvC*
	1	VA, FEP, CT, NA, CTX, LEV, CAZ/CLA, TE, CIP, CEC, FOS, KF, CN, SXT, AZM, MEM	*bla*_TEM_, *bla*_CTX-M1_	*invA*, *stn*, *spvC*
	1	VA, FEP, CT, NA, CTX, LEV, CAZ/CLA, TE, CIP, CEC, FOS, KF, CN, MEM	*bla*_OXA_	*invA*, *stn*
	1	VA, FEP, CT, NA, CTX, LEV, CAZ/CLA, TE, CIP, CEC, FOS, KF, CN, MEM	*bla*_TEM_, *bla*_OXA_	
	1	VA, FEP, CT, NA, CTX, CAZ/CLA, TE, CEC	*bla*_TEM_, *bla*_OXA_	*invA*, *stn*
*Salmonella* Labadi (n = 2)	2	VA, FEP, CT, NA, CTX, LEV, CAZ/CLA, TE, CIP, CEC, FOS, KF, CN, SXT, AZM	*bla*_*TEM*_	*invA*, *stn*
*Salmonella* Apeyeme (n = 2)	1	VA, FEP, CT, CTX, FOS	*bla*_OXA_	*invA, stn*
	1	VA, FEP, CT, CTX	*bla*_CTX-M1_	*invA*, *stn*

VA, Vancomycin; FEP, Cefepime; CT, Colistin; NA, Nalidixic acid; CTX, Cefotaxime; LEV, Levofloxacin; TE, Tetracycline; CAZ/CLA, Ceftazidime/Clavulanic acid; CIP, Ciprofloxacin; CEC, Cefaclor; KF Cephalothin; CN, Gentamicin; SXT, Sulfamethoxazole/Trimethoprim; FOS, Fosfomycin; AZM, Azithromycin; MEM, Meropenem

## Discussion

### Prevalence of *Salmonella* spp. in freshly dressed chicken carcasses

*Salmonella* is a leading foodborne pathogen and has been widely linked to severe foodborne outbreaks cases worldwide. Chicken is the main reservoir of *Salmonella*, which is mainly present in the intestines of live birds [1]. Furthermore, live bird markets are the prime source of *Salmonella* contamination of chicken carcasses. In Egypt, consuming poultry is controlled by cultural legacies, as most Egyptian consumers prefer to go to live poultry shops to select chicken to be slaughtered and receive freshly dressed chicken carcasses. However, most of these shops lack hygienic practices during the slaughtering and processing techniques. In the current study, 357 presumptive *Salmonella* isolates were identified based on conventional cultural morphological characteristics (pink colonies with or without black centers on XLD agar) and biochemical identification methods. The suspected *Salmonella* isolates were tested by PCR targeting *Salmonella* marker gene, the *invA* gene. A total of 129 isolates from 27 native chicken carcasses were confirmed as *Salmonella* with an overall prevalence of 18% (27/150). A similar prevalence rate of *Salmonella* species in chicken carcasses was reported in Egypt by Abd-Elghany et al. [24], who found that 16% of whole chicken carcasses examined were contaminated with *Salmonella* spp. By comparison, a higher prevalence of *Salmonella* in chicken carcasses was reported by other researchers; for instance, *Salmonella* species were detected in 29.4% (50/170) of whole chicken carcasses examined in Egypt [4]. Moreover, 25.1% (156/622) of chicken carcasses in the abattoir environment of Taiwan [2] and 36.4% (138/ 379) of chicken carcasses in two different commercial poultry processing plants in Canada [31] were contaminated with *Salmonella* spp.

Leakage of crop and intestinal contents at the time of the evisceration process are considered the leading sources of poultry contamination by *Salmonella* during slaughtering and processing procedures [32]. Chicken carcasses can be also contaminated with *Salmonella* species due to improper cleaning and sanitation procedures, inadequate chilling and storage temperature, the presence of insects and rodents, and poor personal hygiene in poultry shops [2], besides the contaminated knives, wooden tables, weighing scales, scalding water, chilling tanks, processing equipment such as plucking machines, and cross-contamination from one carcass to another.

### Serotypes of *Salmonella* isolates recovered from chicken carcasses

*Salmonella* serotypes isolated from chicken vary among geographic regions; *S*. Kentucky is the most prevalent serotype in the present study, which is consistent with a previous study conducted by Awad et al. [5], who found that *S*. Kentucky was the dominant serovar among *Salmonella* isolates from retail chicken meat in Egypt with an incidence of 22.6% followed by *S*. Molade with an incidence of 6.5%. Nonetheless, *S.* Typhimurium, *S.* Enteritidis, and *S.* Kentucky were the most prevailing serovars recovered from chicken meat [4, 5, 24, 33].

Among the identified 129 *Salmonella* isolates recovered from freshly dressed native chicken carcasses examined in the present study, 9 were serotyped as *S*. Tsevie at a percentage of 6.9%, which seemed higher than the 3.9% of *S*. Tsevie identified among recovered *Salmonella* isolates from broiler chicken flocks in Qalyubiya Governorate, Egypt [34]. On the other hand, 8 (6.2%), 8 (6.2%), 7 (5.4%), and 6 (4.6%) of the 129 isolates recovered in the current study were serotyped as *S*. Takoradi, *S*. Inganda, *S*. Muenster, *S*. Labadi, respectively; similarly, such serovars were identified among *Salmonella* isolates isolated from chicken carcasses collected from different shops and supermarkets distributed in Mansoura city, Egypt [5, 24]. Interestingly, the least prevalent *Salmonella* serovars in the current study encompass *S*. Giza, *S*. Chester, and *S*. Apeyeme, which were identified only among 3, 3, and 2 of the 129 *Salmonella* isolates, respectively. Previous studies also indicated the identification of *S*. Giza, *S*. Chester, and *S*. Apeyeme in low incidences among *Salmonella* isolates from chicken samples examined in different governorates in Egypt [12, 35, 36], which require more monitoring to protect public health.

### Prevalence, and distribution of virulence genes among *Salmonella* isolated from chicken carcasses

In the current study, all *Salmonella* isolates tested were positive for both *invA* and *stn* genes, while only 31.8% (41/129) of isolates examined were positive for the *spvC* gene. These results are closely similar to those reported by many researchers. For instance, all *Salmonella* serovars isolated from chicken carcasses collected from different shops and supermarkets distributed in Mansoura city, Egypt had both *invA* and *stn* genes [5, 24]. On the other hand, 25.3% (42/166) of *Salmonella* isolates from chicken carcasses examined harbored the *spvC* gene [23], while 39.9% of *Salmonella enterica* serovar Typhimurium recovered from retail raw chickens in China, were positive for the *spvC* gene [37].

The frequency distribution of the *spvC* gene among the 12 different *Salmonella* serovars identified indicated that *S.* Kentucky (n = 12) harbored a high frequency of the *spvC* gene, followed by *S.* Enteritidis (n = 10), *S.* Typhimurium (n = 9), *S.* Tsevie (n = 3), *S*. Takoradi (n = 2),* S*. Muenster (n = 2), *S.* Giza (n = 2), and *S.* Chester (n = 1), while *spvC* gene is absent in *S*. Molade, *S*. Inganda, *S*. Labadi, and *S.* Apeyeme. The *spvC* gene has a prime role in the systemic invasion of the genus in the host cells and could be used as a standard for detecting virulent *Salmonella* strains [14]. Consequently, *Salmonella* isolates from chicken carcasses harboring the *spvC* gene constitute a tremendous public health issue and need a strict monitoring program to avoid the spread of such virulent isolates via food of poultry origin.

### Antimicrobial resistance of *Salmonella* isolates and their classification based on the resistance profile and the multiple antibiotic resistance (MAR) index

The spread and emergence of antimicrobial resistance have been related to the overuse or abuse of antibiotics in animals and humans [38]. In the veterinary field, antibiotics are frequently used as therapeutic, growth promoters, or to enhance the efficiency of food utilization and weight. Multidrug resistance has emerged worldwide as a growing threat to public health threat. Several recent studies concerning the emergence of MDR pathogens from different origins increase the necessity for rationalizing antibiotic usage in veterinary and human medicine [11, 39–43]. *Salmonella* serovars with MDR patterns can produce a variety of multidrug resistance plasmids that harbor resistance genes that mediate resistance to many antimicrobials. Recently, *Salmonella* isolates have undergone several genomic changes and acquired resistance against broad-spectrum cephalosporins through mutated genes that encode for extended-spectrum β-lactamases, hydrolyzing antibiotics with β-lactam rings [6].

The high antimicrobial resistance of *Salmonella* isolates in the current study toward vancomycin, nalidixic acid, and cefepime suggests that these antibiotics are widely used in veterinary medicine. Likewise, *Salmonella enterica* isolates from chicken meat in Turkey exhibited a high resistance rate of 98.8% (83/84) and 89.3 (75/84) towards vancomycin and nalidixic acid, respectively [44].

Surprisingly, 82.2% (106/129) of *Salmonella enterica* isolates in the present study were resistant to colistin; however, colistin is not the drug of choice for treating *Salmonella* infection. A previous study from our laboratory revealed that 39.2% (62/158) of the identified *Salmonella enterica* serovars isolates, recovered from poultry (whole duck, pigeon, and quail carcasses) collected from Mansoura, Egypt, were resistant against colistin [10]. In this study, 51.93% of isolates were resistant to ceftazidime/clavulanic acid, which is a better screening method for the extended-spectrum beta-lactamases (ESBL) in the *Enterobacteriaceae* family [45]. Additionally, 50.4%, 32.6%, and 31% of isolated *Salmonella* strains in the current study were resistant to tetracycline, gentamicin, and sulfamethoxazole/trimethoprim, which are widely used in veterinary medicine as growth promoters, broad-spectrum antibiotic or prophylaxis. In this context, Siriken et al. [44] in Turkey found that 91.6% (77/84), 32.1% (27/84), and 4.8% (4/84) of *Salmonella enterica* isolates from chicken meat were resistant to tetracycline, sulfamethoxazole/trimethoprim, and gentamicin, respectively.

*Salmonella enterica* isolates in the current study revealed a high resistance rate toward the cephalosporin antibiotics encompasses cephalothin, cefaclor, cefotaxime, and cefepime, which constitute a leading global problem as cephalosporins, especially the third- and fourth-generation are the critically important antimicrobials for salmonellosis treatment. Amazingly, the resistance of *Salmonella* isolates against cephalosporins followed the order: cefepime (fourth-generation cephalosporin) > cefotaxime (third-generation cephalosporin) > cefaclor (second-generation cephalosporin) > cephalothin (first-generation cephalosporin), which indicates the improper use and overuse of the third- and fourth-generation cephalosporins in poultry industry. Likewise, most cephalosporin-resistant isolates from poultry in Korea obtained after 2016 were mainly resistant to third- and fourth-generation cephalosporins [46].

Fluoroquinolones are highly effective broad-spectrum antibiotics used mainly for treating human salmonellosis. Due to the wide use of fluoroquinolones in human and animal medicine, high resistance rates of 50.39% and 48.84% were observed against levofloxacin and ciprofloxacin, respectively. By comparison, 63.1% and 44.2% of *Salmonella* isolates from raw chicken meat in Colombia were resistant to ciprofloxacin and levofloxacin, respectively [47]. Moreover, 30.8% (8/26) of *Salmonella enterica* serovars recovered from broiler chickens and chicken carcasses in Egypt were resistant to ciprofloxacin [48]. Fluoroquinolone-resistant *Salmonella* serovars isolated from chicken carcasses are alarming as Fluoroquinolones are the mainstay antibiotics for complicated salmonellosis cases.

*Salmonella enterica* isolates in the current study revealed a low resistance rate of 28.7%, 11.6%, and 10.1% toward fosfomycin, azithromycin, and meropenem, respectively. Fosfomycin displays substantial activity against Gram-negative pathogens involving *Salmonella* spp. The widespread of fosfomycin-resistant *Salmonella* strains constitutes a crucial public health threat as fosfomycin could be an effective treatment option. Likewise, 15.4% of *Salmonella* isolates from broiler chickens and chicken carcasses in Egypt were resistant to azithromycin [48]. On the contrary, 100% of *Salmonella* isolates from broiler carcasses in Colombia were susceptible to imipenem [47]. The emergence of meropenem- and azithromycin-resistant *Salmonella* isolates poses a tremendous public health issue, as they obstruct treatment options for salmonellosis and could increase morbidity and mortality rates.

The average multiple antibiotic resistance (MAR) index for the 129 isolates tested was 0.505, with 82.2% (106/129) of *Salmonella* isolates showing a MAR index above 0.2. A MAR index greater than 0.2 indicates the abuse and excessive use of antimicrobial agents in poultry farms [27]. Therefore, establishing a strict monitoring system to rationalize antimicrobial usage in poultry farms is crucial to protect public health from transferring antimicrobial-resistant bacteria to humans via food of animal origin.

### Distribution of β-lactamase resistance genes among MDR *Salmonella* isolates

Extended-spectrum β-lactamases (ESBLs) confer resistance to third-generation cephalosporins (cefotaxime, ceftriaxone, and ceftazidime) [19]. The most common genetic variant of ESBL is CTX-M [49]. The β-lactamase genes provide resistance to many β-lactam antibiotics, especially cephalosporins (cefotaxime) [10]. A former study revealed that the *bla*_TEM_ was detected in *Salmonella* serovars isolated from broiler chickens and chicken carcasses in Egypt [48]. On the other hand, another study indicated that most of the ESBL-producing *Salmonella* strains (*n* = 9) isolated from diseased and apparently healthy farmed chickens carried *bla*_*TEM*_ and *bla*_*SHV*_ genes, whereas the minority possessed *bla*_*OXA*_ [12]. The emergence of multidrug-resistant (MDR) *Salmonella* species harboring beta-lactamase genes among foods of animal origin highlights the need for surveillance strategies to diminish the usage of antibiotics in veterinary medicines and prevent the transmission of such resistant strains to humans. Consequently, the implementation of the Hazard Analysis Critical Control Point (HACCP) could reduce the hazard of transmission of such pathogenic strains to humans via chicken carcasses.

## Conclusion

The emergence of colistin-, cefepime-, and levofloxacin-resistant *Salmonella* serovars among *Salmonella* isolates from native chicken is worrisome because these antibiotics are the critically important antimicrobials used for treating complicated salmonellosis cases. The current study revealed that native chicken carcasses marketed in Mansoura, Egypt, are contaminated with multidrug-resistant *Salmonella enterica* serovars, which constitutes a tremendous threat to public health. The most predominant *Salmonella* serotypes are *S.* Kentucky, *S*. Enteritidis, *S*. Typhimurium, and *S*. Molade. All *Salmonella* isolates examined harbored both *invA* and *stn* genes, with 31.8% of isolates carrying the *spvC* gene, which is detected only in highly pathogenic *Salmonella* strains. Furthermore, 31 (37.8%) out of 82 cefotaxime-resistant *Salmonella* isolates tested were β-lactamase producers and had at least one of the following β-lactamase resistance genes: *bla*_TEM_, *bla*_CTX-M1,_ and *bla*_OXA_, Therefore, establishing a strict surveillance system to restrict antibiotic use in poultry farms is decisive in protecting public health from transmitting antimicrobial-resistant bacteria to humans via food of poultry origin. Besides, more studies are requested on the emergence and development of antimicrobial-resistant *Salmonella*, which carries many virulent and resistant genes in chicken carcasses.

## Data Availability

No datasets were generated or analyzed during the current study.

## References

[1] Zhang J, Fan X, Ge Y, Yan J, Sun A (2013). Distribution of *Salmonella* paratyphi A *pagC* gene and immunoprotective effect of its recombinant expressed products. Zhejiang da xue xue bao. Yi xue ban J Zhejiang Univ. Med Sci..

[2] Lin CH, Huang JF, Sun YF, Adams PJ, Lin JH, Robertson ID (2020). Detection of chicken carcasses contaminated with *Salmonella enterica* serovar in the abattoir environment of Taiwan. Int J Food Microbiol.

[3] Ketema L, Ketema Z, Kiflu B, Alemayehu H, Terefe Y, Ibrahim M, Eguale T (2018). Prevalence and antimicrobial susceptibility profile of *Salmonella* serovars isolated from slaughtered cattle in Addis Ababa Ethiopia. BioMed Res Int.

[4] Elkenany R, Elsayed MM, Zakaria AI, El-Sayed SAES, Rizk MA (2019). Antimicrobial resistance profiles and virulence genotyping of *Salmonella enterica* serovars recovered from broiler chickens and chicken carcasses in Egypt. BMC Vet Res.

[5] Awad A, Gwida M, Khalifa E, Sadat A (2020). Phenotypes, antibacterial-resistant profile, and virulence-associated genes of *Salmonella* serovars isolated from retail chicken meat in Egypt. World's Vet J.

[6] Eng SK, Pusparajah P, Ab Mutalib NS, Ser HL, Chan KG, Lee LH (2015). *Salmonella*: a review on pathogenesis, epidemiology and antibiotic resistance. Front Life Sci.

[7] Sallam KI, Mohammed MA, Hassan MA, Tamura T (2014). Prevalence, molecular identification and antimicrobial resistance profile of *Salmonella* serovars isolated from retail beef products in Mansoura, Egypt. Food Control.

[8] Sun T, Liu Y, Qin X, Aspridou Z, Zheng J, Wang X, Dong Q (2021). The prevalence and epidemiology of *Salmonella* in retail raw poultry meat in China: a systematic review and meta-analysis. Foods.

[9] CDC. (Centers for Disease Control and Prevention). 2019. Antibiotic resistance threats in the United States. https://www.cdc.gov/drugresistance/pdf/threats-report/2019-ar-threats-report-508.pdf.

[10] Elshebrawy HA, Mahros MA, Abd-Elghany SM, Elgazzar MM, Hayashidani H, Sallam KI (2021). Prevalence and molecular characterization of multidrug-resistant and β-lactamase producing *Salmonella* enterica serovars isolated from duck, pigeon, and quail carcasses in Mansoura Egypt. LWT.

[11] Algammal AM, El-Tarabili RM, Abd El-Ghany WA, Almanzalawi EA, Alqahtani TM, Ghabban H, Al-Otaibi AS, Alatfeehy NM, Abosleima NM, Hetta HF, Badawy GA (2023). Resistance profiles, virulence and antimicrobial resistance genes of XDR. S. Enteritidis S. Typhimurium. AMB Express..

[12] Sabry MA, Abdel-Moein KA, Abdel-Kader F, Hamza E (2020). Extended-spectrum β-lactamase-producing *Salmonella* serovars among healthy and diseased chickens and their public health implication. J Glob Antimicrob Resist.

[13] Fayed AMS, Saad ASA (2021). Effect of microencapsulated allyl-isothiocyanate on survival of *Salmonella* enteritidis and enterotoxin production in ready to eat chicken nuggets. Adv Anim Vet Sci.

[14] Mazurkiewicz P, Thomas J, Thompson JA, Liu M, Arbibe L, Sansonetti P, Holden DW (2008). *SpvC* is a *Salmonella* effector with phosphothreonine lyase activity on host mitogen-activated protein kinases. Mol Microbiol.

[15] Zhao X, Hu M, Zhang Q, Zhao C, Zhang Y, Li L, Liu Y (2020). Characterization of integrons and antimicrobial resistance in Salmonella from broilers in Shandong China. Poult Sci.

[16] Qiao J, Zhang Q, Alali WQ, Wang J, Meng L, Xiao Y, Yang B (2017). Characterization of extended-spectrum β-lactamases (ESBLs)-producing *Salmonella* in retail raw chicken carcasses. Int J Food Microbiol.

[17] Carcione D, Siracusa C, Sulejmani A, Leoni V, Intra J (2021). Old and new beta-lactamase inhibitors: molecular structure, mechanism of action, and clinical use. Antibiotics.

[18] Wong MHY, Yan M, Chan EWC, Biao K, Chen S (2014). Emergence of clinical *Salmonella enterica* serovar typhimurium isolates with concurrent resistance to ciprofloxacin, ceftriaxone, and azithromycin. Antimicrob Agents Chemother.

[19] Bonnet R (2004). Growing group of extended-spectrum β-lactamases: the CTX-M enzymes. Antimicrob Agents Chemother.

[20] USDA/FSIS. 2023. Isolation and Identification of *Salmonella* from Meat, Poultry, Pasteurized Egg, Carcass, and Environmental Sponges. United States Department of Agriculture Food Safety and Inspection Service MLG 4.13. Last accessed

[21] ISO (International Organization for Standardization). 2017. Microbiology of the food chain—horizontal method for the detection, enumeration and serotyping of *Salmonella*—part 1: detection of *Salmonella* spp. ISO standard no. 6579-1. 10.1016/j.ijfoodmicro.2018.03.022 29803313

[22] Grimont PA, Weill FX (2007). Antigenic formulae of the *Salmonella* serovars. WHO Collaborating Center For Reference And Research On Salmonella..

[23] Chiu CH, Ou JT (1996). Rapid identification of *Salmonella* serovars in feces by specific detection of virulence genes, *invA* and *spvC*, by an enrichment broth culture-multiplex PCR combination assay. J Clin Microbiol.

[24] Abd-Elghany SM, Sallam KI, Abd-Elkhalek A, Tamura T (2015). Occurrence, genetic characterization and antimicrobial resistance of *Salmonella* isolated from chicken meat and giblets. Epidemiol Infect.

[25] Huehn S, La Ragione RM, Anjum M, Saunders M, Woodward MJ, Bunge C, Helmuth R, Hauser E, Guerra B, Beutlich J, Brisabois A, Peters T, Svensson L, Madajczak G, Litrup E, Imre A, Herrera S, Mevius D, Newell D, Malorny B (2010). Virulotyping and antimicrobial resistance typing of *Salmonella enterica* serovars relevant to human health in Europe. Foodborne Pathog Dis.

[26] CLSI (Clinical and Laboratory Standards Institute).  (2020). Performance standards for antimicrobial susceptibility testing, M100.

[27] Magiorakos AP, Srinivasan A, Carey RB, Carmeli Y, Falagas ME, Giske CG (2012). Multidrug-resistant, extensively drug-resistant and pandrug-resistant bacteria: an international expert proposal for interim standard definitions for acquired resistance. Clin Microbiol Infect.

[28] Singh S, Yadav AS, Singh SM, Bharti P (2010). Prevalence of *Salmonella* in chicken eggs collected from poultry farms and marketing channels and their antimicrobial resistance. Food Res Int.

[29] Perez F, Hujer AM, Hujer KM, Decker BK, Rather PN, Bonomo RA (2007). Global challenge of multidrug-resistant Acinetobacter baumannii. Antimicrob Agents Chemother.

[30] Ogutu JO, Zhang Q, Huang Y, Yan H, Su L, Gao B, Zhang F (2015). Development of a multiplex PCR system and its application in detection of *bla*_SHV_, *bla*_TEM_, *bla*_CTX-M-1_, *bla*_CTX-M-9_ and *bla*_OXA-1_ group genes in clinical *Klebsiella pneumoniae* and *Escherichia coli* strains. J Antibiot.

[31] Boubendir S, Arsenault J, Quessy S, Thibodeau A, Fravalo P, Thériault WP, Gaucher ML (2021). *Salmonella* contamination of broiler chicken carcasses at critical steps of the slaughter process and in the environment of two slaughter plants: prevalence, genetic profiles, and association with the final carcass status. J Food Prot.

[32] Brizio APDR, Prentice C (2015). Chilled broiler carcasses: a study on the prevalence of *Salmonella*, *Listeria* and *Campylobacter*. Food Res Int.

[33] Khan SB, Khan MA, Ahmad I, ur Rehman T, Ullah S, Dad R, Memon AM.  (2019). Phentotypic, gentotypic antimicrobial resistance and pathogenicity of *Salmonella enterica* serovars typimurium and enteritidis in poultry and poultry products. Microb Pathog.

[34] Abd El-Ghany WA, El-Shafii SS, Hatem ME (2012). A survey on *Salmonella* species isolated from chicken flocks in Egypt. Asian J Anim Vet Adv.

[35] El Sayed A, Abdel-Azeem MW, Sultan S, Abbas A. 2016. Detection of five major pathogenicity islands in *Salmonella* serovars isolated from broiler chicken. Nat Sci. 14(9).

[36] El-Tawab A, Ashraf A, El-Hofy FI, Ammar AM, Nasef SA, Nabil NM (2015). Studies on different *Salmonella* serotypes isolated from poultry in different governorates in Egypt. Benha Med J.

[37] Li K, Ye S, Alali WQ, Wang Y, Wang X, Xia X, Yang B (2017). Antimicrobial susceptibility, virulence gene and pulsed-field gel electrophoresis profiles of *Salmonella* enterica serovar Typhimurium recovered from retail raw chickens China. Food Control.

[38] Vidovic N, Vidovic S (2020). Antimicrobial resistance and food animals: influence of livestock environment on the emergence and dissemination of antimicrobial resistance. Antibiotics.

[39] Abolghait SK, Fathi AG, Youssef FM, Algammal AM (2020). Methicillin-resistant *Staphylococcus aureus* (MRSA) isolated from chicken meat and giblets often produces staphylococcal enterotoxin B (SEB) in non-refrigerated raw chicken livers. Int J Food Microbiol.

[40] Algamma A, Hetta HF, Mabrok M, Behzadi P (2023). Emerging multidrug-resistant bacterial pathogens “superbugs”: A rising public health threat. Front Microbiol.

[41] Algammal AM, Ibrahim RA, Alfifi KJ, Ghabban H, Alghamdi S, Kabrah A, Khafagy AR, Abou-Elela GM, Abu-Elala NM, Donadu MG, El-Tarabili RM (2022). A first report of molecular typing, virulence traits, and phenotypic and genotypic resistance patterns of newly emerging XDR and MDR *Aeromonas veronii* in *Mugil seheli*. Pathogens.

[42] Elbehiry A, Marzouk E, Aldubaib M, Moussa I, Abalkhail A, Ibrahem M, Hamada M, Sindi W, Alzaben F, Almuzaini AM, Algammal AM, Rawway M (2022). *Pseudomonas* species prevalence, protein analysis, and antibiotic resistance: an evolving public health challenge. AMB Express.

[43] Shafiq M, Zeng M, Permana B, Bilal H, Huang J, Yao F, Algammal AM, Li X, Yuan Y, Jiao X (2022). Coexistence of bla NDM-5 and tet (X4) in international high-risk Escherichia coli clone ST648 of human origin in China. Front Microbiol.

[44] Siriken B, Türk H, Yildirim T, Durupinar B, Erol I (2015). Prevalence and characterization of *Salmonella* isolated from chicken meat in Turkey. J Food Sci.

[45] Agarwal S, Chaudhary U (2016). Ceftazidime+ clavulanic acid as a better method of screening extended spectrum beta lactamases (ESBL) in the family *Enterobacteriaceae*. Int J Curr Microbiol Appl Sci.

[46] Jeon HY, Kim YB, Lim SK, Lee YJ, Seo KW (2019). Characteristics of cephalosporin-resistant *Salmonella* isolates from poultry in Korea, 2010–2017. Poult Sci.

[47] Donado-Godoy P, Clavijo V, León M, Arevalo A, Castellanos R, Bernal J, Doyle MP (2014). Counts, serovars, and antimicrobial resistance phenotypes of *Salmonella* on raw chicken meat at retail in Colombia. J Food Prot.

[48] Elkenany RM, Eladl AH, El-Shafei RA (2018). Genetic characterisation of class 1 integrons among multidrug-resistant *Salmonella* serotypes in broiler chicken farms. J Glob Antimicrob Resist.

[49] Paterson DL, Bonomo RA (2005). Extended-spectrum beta-lactamases: a clinical update. Clin Microbiol Rev.

